# Enhancing the Shelf Life of Food Products: Strategies, Challenges, and Innovations

**DOI:** 10.3390/foods14234034

**Published:** 2025-11-25

**Authors:** Alessandro Bianchi, Francesca Venturi

**Affiliations:** 1 Department of Agriculture, Food and Environment, University of Pisa, Via del Borghetto 80, 56124 Pisa, Italy; francesca.venturi@unipi.it; 2 Department of Pharmacy, University of Pisa, Via Bonanno Pisano 12, 56126 Pisa, Italy; 3 Interdepartmental Research Centre “Nutraceuticals and Food for Health”, University of Pisa, Via del Borghetto 80, 56124 Pisa, Italy

## 1. Introduction

The concept of shelf life is fascinating because it combines scientific issues with economic, governmental, and consumer considerations [1]. The latter factors, which can drastically differ from nation to nation, are dynamic and are subject to change in response to shifts in society, particularly in terms of consumer preferences and needs [2,3]. In the face of increasing global demand, shifting consumer expectations, and pressing sustainability goals, enhancing the shelf life of food products while maintaining their overall quality remains a central challenge in food science and technology [2,3,4].

Shelf life is not merely a label datum: it encapsulates complex interactions among microbial ecology, physicochemical stability, packaging systems, supply chain logistics, and consumer behavior [2]. In recent decades, the paradigm has shifted from relying on synthetic preservatives and high-intensity treatments toward milder, integrative, and sustainable strategies that better preserve nutritional and sensory attributes while reducing waste and aligning with the “clean label” movement [5,6]. Despite these advances, major gaps remain in bridging laboratory innovation and real-world applicability, in quantifying environmental tradeoffs, and in understanding dynamic microbial ecosystems under storage [7].

To obtain reliable shelf life data, it is essential to refer to a systematic and reliable approach. To achieve this aim, a comprehensive shelf life study can be divided into three fundamental steps. First, the identification of the most critical chemical, physical, or biological events leading to product quality depletion is required, followed by the definition of the relevant acceptability limits. Second, the selected quality indicators are monitored as a function of time under storage conditions that mimic foreseeable conditions (real-time shelf life testing) or under accelerated environments that enhance deteriorative reactions (accelerated shelf life testing, ASLT). Finally, experimental data are modeled to obtain an estimation or prediction of shelf life [1,7,8,9]. Real-time testing is especially suitable for perishable foods with short quality decay periods, while accelerated testing is more appropriate for microbiologically stable foods such as ambient and frozen products, where oxidative reactions dominate quality depletion [5,9,10].

Achieving the desired shelf life is a powerful driver for product and packaging innovation. In particular, packaging technologies have played a crucial role in extending product life and maintaining quality [11]. Modified atmosphere packaging (MAP) exemplifies this progress, relying on oxygen exclusion to limit oxidative rancidity and microbial spoilage [12,13,14,15]. Beyond MAP, a wide range of active and intelligent packaging systems have emerged to interact dynamically with the packaged food and its environment. ‘Smart’ packaging encompasses both active and intelligent forms: active materials may release or absorb substances (e.g., ethanol vapors to inhibit microbial growth), while intelligent devices—such as inexpensive colorimetric tags or RFID (Radio Frequency Identification) sensors—enable communication across the supply chain to ensure quality and safety [16,17]. Recent studies have also explored biobased smart materials incorporating natural pigments derived from plant products or food by-products—such as anthocyanins, curcumin, betalains, carotenoids, tannins, and chlorophyll—as visual indicators of spoilage [18,19,20,21,22,23]. These pigments, sensitive to volatile amines and organic acids released during deterioration, undergo visible color changes, allowing real-time, non-invasive monitoring of freshness and safety [20].

Besides packaging innovations, emerging food processing technologies have redefined shelf life enhancement paradigms. At the turn of the century, novel non-thermal technologies such as high hydrostatic pressure (HHP), pulsed electric fields (PEF), ultrasound (US), and high-pressure carbon dioxide (HPCD) processing have gained attention due to their ability to inactivate microorganisms while preserving the sensory and nutritional characteristics of foods [24,25,26,27]. These methods offer reduced thermal degradation, energy efficiency, and the possibility of combination with traditional treatments for optimized microbial and enzymatic control [28]. Integrating non-thermal methods with active packaging systems represents a promising direction for the future, potentially leading to synergistic effects in shelf life extension without compromising the “fresh-like” appeal of minimally processed foods [29,30,31].

Another rapidly growing field involves natural preservatives and antimicrobial compounds derived from plants. Chemical preservatives have long been used to control spoilage and pathogenic microorganisms, but growing consumer demand for minimally processed and additive-free products has shifted the focus toward natural alternatives [32,33,34,35]. Plant-derived bioactive compounds—including phenolics, alkaloids, flavonoids, steroids, and terpenes—exhibit significant antibacterial and antioxidant properties, providing a natural means of food protection during storage [36,37,38,39,40]. Essential oils, in particular, have gained traction for their strong antimicrobial activity, though challenges remain in their controlled release, sensory impact, and stability during processing and storage [39,41,42].

Looking ahead, the integration of predictive modeling, real-time sensing, and sustainable preservation strategies is expected to transform shelf life management [43]. Data-driven approaches, powered by artificial intelligence and digital twins, can simulate degradation kinetics under varying storage and distribution scenarios, improving the precision of shelf life prediction [39,44,45]. Moreover, advances in biodegradable and compostable packaging materials, coupled with circular economy principles, are pushing the field toward eco-designed shelf life systems that minimize waste and environmental impact. These multidimensional efforts are essential to support a holistic, science-based, and sustainability-oriented framework for shelf life extension and monitoring [43,44,46,47,48].

The present Special Issue was conceived to address these challenges. The editorial vision aimed to gather contributions that transcended incremental improvements, emphasizing interdisciplinary approaches that combine formulation, processing, packaging, microbiological control, and environmental sustainability. Across its contributions, the Issue encompasses diverse food matrices (meat, fish, fruits, berries, minimally processed vegetables, and dairy) and technological strategies (antimicrobials, coatings, mild processing, active and intelligent packaging, and life-cycle assessment). In the following sections, we synthesize the main insights, identify persisting research gaps, and propose a forward-looking agenda for future innovations in shelf life science.

## 2. Key Contributions and Their Implications

This Special Issue was conceived to gather original research and review papers addressing innovative strategies for the control, analysis, and preservation of microorganisms in foods, with a particular focus on natural antimicrobials, sustainable preservation technologies, valorization of food by-products, and quality–safety interactions during processing and storage. The eleven contributions collected in this Issue exemplify the breadth of current research, spanning from traditional food safety control to novel materials, environmental assessments, and sensory optimization. Below, a brief overview of each contribution is presented to encourage the reader to explore the full papers.

A central piece in this Issue is the review by Rabbani and collaborators (Contribution 8) on the effect of heat pasteurization and sterilization on milk. The authors comprehensively discuss how conventional thermal treatments—while ensuring microbial safety—unavoidably modify protein conformation, flavor, and micronutrient integrity, thus compromising quality. The review also highlights the growing interest in emerging non-thermal and combined technologies, such as pulsed electric fields and high-pressure processing, as promising tools to maintain both safety and nutritional value.

Building on this technological perspective, Dermesonlouoglou and colleagues (Contribution 6) present a hybrid preservation approach combining pulsed electric fields (PEF) and osmotic dehydration followed by modified-atmosphere packaging of fresh-cut and fried potatoes. This multi-step protocol effectively slows down microbial proliferation and oxidation, thereby illustrating the strength of hurdle technology, where mild complementary treatments act synergistically to preserve food quality without compromising texture or flavor.

In a related direction, Faisal and co-authors (Contribution 5) investigated an edible coating composed of turmeric extract and liquid smoke obtained from oil-palm empty fruit bunches, applied to mackerel filets. The coating significantly delayed microbial spoilage and maintained sensory acceptability for up to 48 h at room temperature, offering a low-cost preservation strategy particularly suited for warm-climate regions where cold storage is limited. Extending this concept to fruit products, Mari and collaborators (Contribution 7) integrated a Life Cycle Assessment (LCA) into a process combining osmotic dehydration and edible coatings for berries. Their work quantifies the environmental trade-offs of such innovative technologies, identifying energy use and coating formulation as major hotspots, and providing an evidence-based framework for the sustainable design of food preservation methods.

Natural antimicrobials and valorized ingredients are also at the core of several other contributions. Kačániová and co-authors (Contribution 1) demonstrated that the essential oil of *Eugenia caryophyllus* (clove) effectively inhibits *Salmonella enterica* and biofilm formation, improving the microbial safety and shelf life of sous-vide deer meat. Similarly, Tayel and collaborators (Contribution 9) evaluated dill (*Anethum graveolens*) essential oil as a preservative for fish filets stored under refrigeration, finding a substantial reduction in spoilage microorganisms and oxidative degradation, leading to longer freshness retention.

The incorporation of bioactive natural extracts into functional materials is another emerging trend showcased in this Issue. García-Juárez and colleagues (Contribution 3) developed gelatin nanoparticles loaded with bitter orange (*Citrus aurantium*) peel extract, revealing remarkable antioxidant and antibacterial activities, which highlight their potential as active components for edible coatings or packaging aimed at extending shelf life and reducing synthetic additives.

Complementing these innovations, Augustyńska-Prejsnar and co-authors (Contribution 4) investigated microbial and sensory quality changes in broiler chicken breast meat during refrigerated storage. Their findings provide valuable data on the relationship between microbial growth dynamics and sensory deterioration, supporting the establishment of realistic shelf life and quality criteria for fresh poultry.

The valorization of agro-industrial by-products was further addressed by D’Arrigo and collaborators (Contribution 2), who incorporated a red grape pomace ingredient—stabilized through blanching and high-pressure processing—into traditional dry-cured *salchichón* sausages. The inclusion enhanced antioxidant capacity and limited lipid and protein oxidation, offering a sustainable alternative to synthetic additives such as nitrites, while maintaining desirable sensory characteristics.

A different but complementary strategy was explored by Rodrigues and colleagues (Contribution 10), who examined dietary supplementation of Nile tilapia (*Oreochromis niloticus*) with the green alga *Chlorella pyrenoidosa*. The enriched diet improved the antioxidant stability and microbial resistance of the filets during refrigerated storage, demonstrating how nutritional interventions at the farming stage can enhance post-harvest quality and shelf life.

Finally, Panzani and co-authors (Contribution 11) compared traditional versus controlled drying methods for chestnuts (*Castanea sativa*), showing that controlled drying preserved more favorable chemical and aromatic profiles, resulting in superior sensory quality of chestnut flour. While not directly antimicrobial, this study reinforces the Issue’s overarching theme—that innovative, controlled processing methods can simultaneously safeguard quality, extend shelf life, and align with sustainability goals.

Collectively, these contributions underscore the multifaceted nature of modern food preservation, spanning from molecular to environmental scales. They highlight how natural compounds, gentle physical technologies, valorized materials, and life cycle thinking converge toward the shared objective of achieving safe, high-quality, and sustainable foods, thus offering valuable guidance for both researchers and industry practitioners.

## 3. Future Directions and Research Agenda

Although remarkable progress has been achieved in the field, several aspects still warrant further investigation to ensure that advances translate effectively from research to real-world applications. Scaling up experimental results remains a key challenge, as most studies are performed under controlled laboratory conditions and must now be validated on a pilot or industrial scale. Similarly, shelf life optimization should be approached holistically, considering its interplay with the supply chain, cold-chain logistics, and consumer behavior. The development of reliable predictive models that integrate microbial kinetics, packaging performance, and storage conditions is another promising yet evolving area.

Moreover, future research would benefit from a more comprehensive evaluation of sustainability parameters—including energy, water, and carbon impacts—supported by life cycle and techno-economic analyses. Continued attention is also needed to ensure that novel materials, coatings, and active systems comply with regulatory frameworks and meet consumer expectations. Looking ahead, efforts should focus on hybrid smart packaging systems capable of dynamic monitoring and sensor feedback, as well as on material innovation toward biodegradable and functional solutions. The integration of digital tools such as data-driven models and digital twins could further enhance predictive accuracy and process optimization, paving the way for a more efficient, sustainable, and consumer-centered food packaging ecosystem.

## 4. Conclusions

This Special Issue has sought to bring together innovation, scientific rigor, and sustainability within the broad domain of food shelf life extension. The contributions collected herein clearly show that the future of the field lies not in single “silver bullet” technologies, but rather in smartly integrated, system-level strategies that combine mild processing, intelligent packaging, ecological assessment, and real-world validation.

Nevertheless, the journey toward truly sustainable and resilient food preservation is far from complete. It is hoped that this closing editorial will inspire further cross-disciplinary collaboration, motivating researchers to bridge the gap between laboratory experimentation and pilot- or industrial-scale implementation. Equally, it should encourage the exploration of new research pathways that connect microbiology, materials science, data analytics, and sustainability assessment.

Readers are invited to engage with the articles in this Special Issue, identify emerging synergies, and contribute to advancing the shared goal of safe, high-quality, and environmentally responsible food systems for the future.

## Data Availability

Not applicable.
